# Comparison of empathy with patients between first-year and last-year medical students of Tehran University of Medical Sciences

**DOI:** 10.1186/s12909-021-02897-0

**Published:** 2021-08-30

**Authors:** Reyhane Hizomi Arani, Zohrehsadat Naji, Ali Moradi, Seyed Vahid Shariat, Sara Mirzamohamadi, Payman Salamati

**Affiliations:** 1 Sina Trauma and Surgery Research Center, Tehran University of Medical Sciences, P.O. Box: 1136746911, Tehran, Iran; 2 Young Researchers and Elites Club, Science and Research Branch, Islamic Azad University, Tehran, Iran; 3 Occupational Health and Safety Research Center, Hamadan University of Medical Sciences, Hamadan, Iran; 4 Mental Health Research Center, Psychosocial Health Research Institute, Iran University of Medical Sciences, Tehran, Iran; 5 School of Behavioral Sciences and Mental Health (Tehran Institute of Psychiatry), University of Medical Sciences, Tehran, Iran

**Keywords:** Empathy, Communication skills, Ethics/attitudes, Professionalism, Curriculum, Behavioral sciences

## Abstract

**Purpose:**

This study was conducted to assess the developmental factors of empathy among medical students of Tehran University of Medical Sciences (TUMS).

**Methods:**

To assess the empathy levels, 42 first-year and an equal number of last-year medical students were randomly selected. They answered a questionnaire including the medical student version of the Jefferson Scale, demographic, and some related variables. Statistical analyses were performed using the Chi-square test, T-test, univariate, and multivariate regressions.

**Result:**

The study population consisted of 51 (60.7 %) men and 33 (39.3 %) women with a mean (SD) age of 22.24 (4.10) years. The Jefferson score was 110.19 ± 13.61 and 103.52 ± 20.00 in first- and last-year medical students, respectively. Moreover, medical students who completely passed at least one of the considered empathy courses of the TUMS curriculum had higher empathy scores compared to their counterparts (109.83 ± 15.54 vs. 103.68 ± 19.02). There was no significant association between empathy and gender, self-experience of illness, marital status, family history of chronic disease, and parents’ education (all P-values > 0.05). After adjusting for the effects of confounders, the empathy scores were significantly associated with the academic year level (*p* = 0.04), level of interest in medicine (*p* = 0.001), and passing the empathy courses (*p* = 0.04).

**Conclusions:**

The data provided from a top Iranian medical school indicated that the academic year level, level of interest in medicine, and passing the empathy courses were significantly associated with the empathy level. Further studies are recommended.

**Supplementary Information:**

The online version contains supplementary material available at 10.1186/s12909-021-02897-0.

## Introduction

Empathy is the capacity to understand or feel others and the art of seeing the world from others’ perspectives [1]. Like any other skills, empathy requires learning and training. Some countries consider empathy training as part of the medical curriculum related to the subject of professional ethics (professionalism) [2], which teaches the students how to communicate with the patient and their companions [3]. It should be considered that empathy tends to deviate negatively in the absence of targeted programs [4, 5].

In recent years, early professional training has been added to the Iranian undergraduate medical curriculum besides basic sciences to encourage multidimensionality and altruism.

The curriculum of Tehran University of Medical Sciences (TUMS) consists of three types of courses that can improve and teach empathy directly or indirectly: First, longitudinal weekly courses presented in the first three years at the faculty, with three extra sessions exactly before entering the hospitals including panels for personal growth such as decision-making, leadership, teamwork, and communication skills in the hospital context. Second, medical ethics classes including patients’ rights, confidentiality of patients’ secrets, respecting their authority, and empathizing with them upon sharing news [6]. Third, religious and spiritual courses that encourage self-devotion and a good relationship with human beings based on Islamic teachings.

Numerous questionnaires have been designed for measuring empathy around the world. In the early 21st century, researchers at Jefferson University, USA developed a questionnaire to evaluate the physicians’ empathy [7].

The importance of empathy as an essential skill for medical students in clinical practice is well known; however, its associated factors remain poorly understood. There are some hypotheses that the level of empathy in medical students decreases with an increase in the academic year due to a sudden increase in workload, increased patient encounter, *and* decreased job satisfaction [8, 9]. On the other hand, there are controversial and inconclusive studies about the factors affecting the empathy level, such as gender, level of interest in medicine, and passing the empathy courses. Hence this study was conducted to compare empathy with patients between first- and last-year medical students (juniors and seniors) of Tehran University of Medical Sciences (TUMS) and its associated factors.

## Methods

### Participants

The present cross-sectional study was conducted at TUMS in 2020. One hundred and twenty medical students enrolled in TUMS after passing a highly competitive entrance exam. As TUMS has a high quality of education among Iranian universities with no tuition fee, it has many volunteers from all over the country. The TUMS curriculum contains 2.5 years for basic sciences, one year for pathophysiology and each organ’s disease, two years of externship, and finally 1.5 years of internship. In this study, first, international students were excluded, so all of the participants were originally Iranian and fluent in the Persian language. Furthermore, guest students who were temporarily studying at this university were also excluded to have a more homogeneous population. Then, 42 first-year and an equal number of last-year medical students were randomly selected using a table of random numbers.

### Data sampling

To measure empathy in medical students, the medical student version of the Jefferson scale was employed. The questionnaire was developed to evaluate empathy in medical students and contains 20 items on a Likert scale. The participants give 1 to 7 points to each item, depending on to what extent they agree with the item. A score of “one” indicates disagreement and a score of “seven” indicates maximum agreement. The English version of the questionnaire is provided as a supplementary file named “Additional file 1”. The validity and reliability of the original questionnaire was examined and confirmed in several studies. Its test-retest reliability coefficient and Cronbach’s alpha coefficient were 0.65 and 0.81, respectively [10]. The Persian version of this questionnaire was studied by Shariat et al., and its Cronbach’s alpha and the test-retest reliability coefficient were 0.79 and 0.95, respectively [11]. The same translated version was used in this study. Written informed consent was obtained from all subjects and they were also assured that their personal information would remain confidential. The protocol of the study complied with TUMS guidelines for manuscript submission.

### Definition of terms

Demographic characteristics and other Dara including medical students’ degree, age, gender and marital status were collected. Moreover, there were some questions about the number of times (s)he was hospitalized or referred to a specialist per year until 2the time of the study, length of hospital stay, parents’ education, empathy courses, history of chronic disease in the family, and level of interest in medicine.

Passing empathy courses was defined as passing at least one of the curriculum empathy courses completely including “longitudinal”, “medical ethics”, and “religious” courses which explained above.

### Statistical analysis

The number of the participants in each group (42) was estimated using the routine sample size calculation for comparing two averages (Z_1-α/2_= 1.96 & Z_1-β_= 0.84, S1 = 13.3, S2 = 15.1, µ1 = 105.5, µ2 = 96.8) according to a study by Shariat et al. [11]. The statistical package SPSS 21 for Windows was used for analysis. Data are presented as mean ± standard deviation for continuous and number (%) for dichotomous variables. Chi-square and T-test were applied for group comparison as appropriate. The stepwise multivariable regression analysis was employed for controlling the effects of confounding factors to find the potential predictors of empathy scores among our participants including academic year level, gender, categories of marital status, levels of interest in medicine, fathers’ education and passed empathy courses using dummy coding method. P-values < 0.05 were considered statistically significant.

## Results

Eighty-four participants including 51 (60.7 %) men and 33 (39.3 %) women with a mean (SD) age of 22.24 (4.10) years were enrolled in this study.

Table 1 shows the absolute and relative frequency of some related characteristics of the participants. Generally, most of the participants were single (91.7 %) and their parents’ education levels were higher than high school diploma (79.8 % of the fathers and 70.2 % of the mothers). About 58.3 % of the subjects passed empathy courses. At least a history of hospitalization, a family history of chronic diseases, and a history of referral to a specialist was found in 38.8 %, 48.8 %, and 10.7 % of the participants, respectively. Statistical tests showed significant differences in marital status and history of chronic disease in the family between the two groups, while the two groups were identical in terms of other variables.

**Table. 1:** Some characteristics of the participants

	First-year		Last-year		Total		P-value
	Number	percent	Number	percent	Number	percent	
Marital status0.01							
- Single	42	100	34	81.0	76	90.5	
- Divorced	0	0	1	2.4	1	1.2	
- Married	0	0	7	16.7	7	8.3	
Father’s education0.74							
- Ph.D.	12	28.6	10	23.8	22	26.2	
- Master’s degree	8	19.0	6	14.3	14	16.7	
- Bachelor’s	16	38.1	15	35.7	31	36.9	
- High school diploma	5	11.9	9	21.4	14	16.7	
- Lower education level	1	2.4	2	4.8	3	3.6	
Mother’s education0.29							
- Ph.D.	8	19.0	5	11.9	13	15.5	
- Master’s degree	10	23.8	6	14.3	16	19.0	
- Bachelor’s degree	11	26.2	19	45.2	30	35.7	
- High school diploma	11	26.2	8	19.0	19	22.6	
- Lower education level	2	4.8	4	9.5	6	7.1	
Passing empathy courses 0.82							
- Yes	24	57.1	25	59.5	49	58.3	
- No	18	42.9	17	40.5	35	41.7	
Hospitalization history0.36							
- Yes	14	33.3	18	42.9	32	38.1	
- No	28	66.7	24	57.1	52	61.9	
History of chronic diseases in the family0.01							
- Yes	15	35.7	26	61.9	41	48.8	
- No	27	64.3	16	38.1	43	51.2	
History of referral to a specialist0.69							
- Yes	5	11.9	4	9.5	9	10.7	
- No	37	88.1	38	90.5	75	89.3	

Table 2 shows the comparison of the mean score of empathy among the students according to the academic year level (first-year vs. last-year students), gender, and passing empathy courses (*p* = 0.07, *p* = 0.32, and *p* = 0.06, respectively). The mean score of empathy was higher in first-year students, female students, and students who passed empathy courses compared to their counterparts; however, univariate analysis showed no significant differences.

**Table. 2:** Comparison of mean score of empathy among students

	Mean	Variance	Mean difference (95 % CI)	P-value
Academic year level			6.66 (0.76–14.09)	0.07
- First-year	110.19	13.61		
- Last-year	103.52	20.00		
Gender			3.85 (3.84–11.56)	0.32
- Female	108.37	13.89		
- Male	104.51	21.63		
Passing empathy courses			7.15 (0.36–14.66)	0.06
- Yes	109.83	15.54		
- No	102.68	19.02		

The final multivariate model of the factors affecting empathy scores is presented in Table 3. The academic year level, gender, marital status, father’s education level, and passing empathy courses (as categorical variables) and level of interest in medicine (as an ordinal variable) were entered in the final model. After adjustment for confounders, the empathy score was found to be significantly associated with the academic year level (*p* = 0.04), level of interest in medicine (*p* = 0.001), and passing empathy courses (*p* = 0.04).

**Table 3:** Final model of factors influencing empathy scores

	Unstandardized Coefficients		Standardized Coefficients	P-value
Model	**B**	**Std. Error**	**Beta**	
Constant	6.94	2.43		0.005
Academic year level				
1- First-year				
2- Last-year	-1.88	0.94	-0.21	0.04
Gender				
1- Women				
2- Men	-0.37	0.95	-0.04	0.69
Marital status				
1- Not married				
2- Divorced	1.11	4.32	0.01	0.873
3- Widowed	1.32	4.00	0.03	0.742
4- Married	2.67	1.68	0.16	0.117
Interested in medicine				
1- Very high				
2- High	-0.81	1.05	-0.81	0.446
3- Moderate	-3.00	1.10	-0.31	0.008
4- Low	-15.64	4.05	-0.38	< 0.001
5- Very low	-17.71	4.89	-0.39	< 0.001
Father’s education				
1- Ph.D.				
2- Master’s degree	-0.03	1.33	-0.003	0.977
3- Bachelor’s degree	0.67	1.12	0.07	0.548
4- High school diploma	1.71	1.38	0.14	0.219
5- Lower education level	2.68	2.43	0.11	0.273
Passing empathy courses				
1- Yes				
2- No	-1.84	0.39	-0.20	0.04

Stepwise multivariable regression analysis adjusted for academic year level (reference: first year), gender (reference: women), categories of marital status (reference: not married), levels of interest in medicine (reference: very high), fathers’ education (reference: Ph.D.) and passed empathy courses (reference: yes).

## Discussion

The present study assessed medical students’ empathy with patients among juniors and seniors at TUMS. The mean score of empathy was (106.85 ± 16.80) in our study using the Jefferson Scale. After adjusting for the effects of confounders, the empathy scores were significantly associated with the academic year level, interest in medicine, and passing empathy courses. There was no significant association between the empathy score and gender, self-experience of illness, marital status, family history of chronic disease, and parents’ education level.

The results showed that empathy decreased with an increase in the years of study in the medical school, which was inconsistent with the results of a study by Hojat et al. [12, 13] a multicenter study [14], and a systematic review [15]. However, some other studies found contrary results [16, 17]. Overall, there is no clear, conclusive, and general international trend about changes in the students’ empathy throughout medical education [18, 19]. There are several possible explanations for this decline. It may be due to the long duration of educational programs, limited bedside interactions, and students’ negative experiences in medical school apart from different baseline characteristics of students applying to medical schools [20]. Another explanation is that the stressful perspective of medical education, such as long work hours and sleep deprivation [21, 22], may lead to higher emotional exhaustion, depression, depersonalization, and burnout in the final years of medical school. A systematic review indicated a large burden of profession-related burnout (range: 7.0–75.2 %) among medical students in different sections of medical education [23], which may be characterized by reduced efficiency and motivation, exhaustion, restlessness, and tension [24]. In this regard, Moir et al. reported that anxiety, depression, and stress might reduce the signal rate of mirror neurons, contributing to the ability of understanding others and empathize [25].

The present study showed that empathy tended to be lower in students that were less interested in medicine. It may be because of the association of empathy and factors affecting the level of interest in medicine, such as positive social interactions, quality of life, and life satisfaction [15]. Furthermore, our study demonstrated that the empathy score was significantly higher in medical students who passed empathy courses compared to students who did not. This finding is in line with a systematic review suggesting that educational interventions can be effective in maintaining and enhancing empathy among undergraduate medical students [26].

To the best of our knowledge, no study has investigated the association between empathy and biographical experiences (such as the number of hospitalizations, referral to specialists per year, the length of hospitalization, and having a chronic disease in the family). It is possible that medical students selfishly regulate their relationships with others, taking into account their interests and comforts. They may reduce their empathy as a self-defense reaction in the face of traumatizing work.

The results showed that the parents’ education level had no significant impact on medical students’ empathy; however, another study found that empathy was significantly associated with the mother’s level of education, a satisfactory relationship with the mother, and household income [27]. Based on family structure in Iran, people can live a long time with their family which may lead to cultural and behavioral influences [28]. However, the point is that the level of empathy is not necessarily related to social class and level of education. Interestingly, a higher social class is predictive of increased unethical behavior according to some studies [29, 30].

Unlike some studies, [31–33] the present study found no relationship between gender and the mean empathy score. It seems that the gender role may decrease in empathic dispositions in the relationship with others in the medical care context of Iran.

It is worth noting that marital status and history of chronic diseases in the family were significantly higher in last-year medical students compared to first-year ones, which could be due to age. Indeed, ageing increases the chance of marriage. In addition, as time passes, parents become older and prone to chronic diseases.

Based on the results, it is recommended that the level of interest and emotional desires should be evaluated upon starting medical education. These findings could help educational system to make decisions for maintaining the enthusiasm of medical students, not to give way to motiveless, fatigue, and decreased vigor-activity. More research is recommended using longitudinal and multi-institutional studies.

## Strengths and Limitations

A strength of the present study was the inclusion of different baseline characteristics. In addition, we used a random sample, which is important for enhancing the generalizability of the results and mitigating selection bias. A limitation was that it was a cross-sectional study with no longitudinal cohort follow-up. Another limitation was its single-center design, which limits the generalizability of results to other people. However, this limitation can be justified by the fact that TUMS is the best medical university in Iran with high heterogeneity regarding the demographic characteristics of students from different regions of Iran.

## Conclusions

The data from a top Iranian medical school showed a lower empathy score compared to some studies conducted in western countries such as the US [13] and Brazil [31]. A significant positive association was found between medical students’ empathy and the level of interest in medicine and passing empathy courses; however, empathy declined significantly in higher academic year levels.

## Supplementary information

**Additional file 1.**

## Data Availability

The datasets used and analyzed in the current study are available from the corresponding author upon request.
