# Supplementary material for: Comparison of empathy with patients between first-year and last-year medical students of Tehran University of Medical Sciences
Source: BMC Med Educ. 2021 Aug 30;21:460. doi: 10.1186/s12909-021-02897-0 (PMC8406781; doi:10.1186/s12909-021-02897-0)
Supplement: Supplementary file 1 — Additional file 1. [file 12909_2021_2897_MOESM1_ESM.doc]

**Comparison of empathy with patients between first-year and last-year medical students at Tehran University of Medical Sciences**

Running Head: Comparison of empathy among medical students

Reyhane Hizomi Arani^1^, Zohrehsadat Naji^2^, Ali Moradi^3^, Seyed Vahid Shariat^4,5^, Sara Mirzamohamadi ^1^, Payman Salamati ^1^

1-Sina Trauma and Surgery Research Center, Tehran University of Medical Sciences, Tehran, Iran.

2- Young Researchers and Elites Club,

Science and Research Branch, Islamic Azad University, Tehran, Iran.

3- Occupational Health and Safety Research Center, Hamadan University of Medical Sciences, Hamadan, Iran

4- Mental Health Research Center, Psychosocial Health Research Institute, Iran University of Medical Sciences, Tehran, Iran.

5- School of Behavioral Sciences and Mental Health (Tehran Institute of Psychiatry), Iran University of Medical Sciences, Tehran, Iran.

Corresponding Author:

Payman Salamati, M.D.

Professor of Social Medicine

Sina Trauma and Surgery Research Center, Tehran University of Medical sciences, Tehran, Iran

P.O. Box: 1136746911

Phone: +98 21 66348500

Fax: +98 21 66348553

Cell: +98-912-3158950

Email: [psalamati@tums.ac.ir](mailto:psalamati@tums.ac.ir)

**Jefferson Scale of Physician Empathy (S-Version R)**

**Name:________________________________Sex :_________________ SS#: _________________________**

***Instructions:***  Please indicate the extent of your agreement or disagreement with *each* of the following statements by writing the appropriate rating number on the underlined space provided before each statement. Please use the following 7-point scale (*a higher number* *on the scale indicates more agreement):*

***1-------2-------3-------4-------5-------6-------7***

***Strongly Disagree Strongly Agree***

1. __ Physicians’ understanding of their patients’ feelings and the feelings of their patients’ families does not influence medical or surgical treatment.
2. __ Patients feel better when their physicians understand their feelings.
3. __ It is difficult for a physician to view things from patients’ perspectives.
4. __ Understanding body language is as important as verbal communication in physician-patient relationships.
5. __ A physician’s sense of humor contributes to a better clinical outcome.
6. __ Because people are different, it is difficult to see things from patients’ perspectives.
7. __ Attention to patients’ emotions is not important in history taking.
8. __ Attentiveness to patients’ personal experiences does not influence treatment outcomes.
9. __ Physicians should try to stand in their patients’ shoes when providing care to them.
10. __ Patients value a physician’s understanding of their feelings which is therapeutic in its own right.
11. __ Patients’ illnesses can be cured only by medical or surgical treatment; therefore, physicians’ emotional ties with their patients do not have a significant influence in medical or surgical treatment.
12. __ Asking patients about what is happening in their personal lives is not helpful in understanding their physical complaints.
13. __ Physicians should try to understand what is going on in their patients’ minds by paying attention to their non-verbal cues and body language.
14. __ I believe that emotion has no place in the treatment of medical illness.
15. __ Empathy is a therapeutic skill without which the physician’s success is limited.
16. __ Physicians’ understanding of the emotional status of their patients, as well as that of their families is one important component of the physician-patient relationship.
17. __ Physicians should try to think like their patients in order to render better care.
18. __ Physicians should not allow themselves to be influenced by strong personal bonds between their patients and their family members.
19. __ I do not enjoy reading non-medical literature or the arts.
20. __ I believe that empathy is an important therapeutic factor in medical treatment.

© Jefferson Medical College, 2000.
